# Racial Gap in Household Income Explains Black-White Disparities in the Intergenerational Transmission of Educational Attainment

**DOI:** 10.31586/ojer.2024.962

**Published:** 2024-07-12

**Authors:** Shervin Assari, Hossein Zare

**Affiliations:** 1 Department of Internal Medicine, Charles R. Drew University of Medicine and Science, Los Angeles, CA, United States; 2 Department of Family Medicine, Charles R. Drew University of Medicine and Science, Los Angeles, CA, United States; 3 Department of Urban Public Health, Charles R. Drew University of Medicine and Science, Los Angeles, CA, United States; 4 Marginalization-Related Diminished Returns (MDRs) Center, Los Angeles, CA, United States; 5 Department of Health Policy and Management, Johns Hopkins Bloomberg School of Public Health, Baltimore, MD, United States; 6 School of Business, University of Maryland Global Campus (UMGC), College Park, MD, United States

**Keywords:** Ethnic Groups, Racism, Young Adult, Incarceration, Social Mobility

## Abstract

**Background:**

Racial disparities in educational outcomes persist in the United States, with Black individuals experiencing lower levels of educational attainment and a higher rate of school disciplinary actions compared to their White counterparts. Parental education is a known predictor of offspring educational attainment, but its effects may vary by race. Understanding the role of household income in mediating these effects is crucial for developing targeted policy interventions to reduce educational inequalities.

**Objectives:**

This study aimed to examine the role of household income in mediating the differential effects of maternal education on two youth educational outcomes (educational attainment by age 22 and school disciplinary action) in Black and White families.

**Methods:**

Data were drawn from the 22 years of follow-up of the Future of Families and Child Wellbeing Study (FFCWS), a longitudinal study following a cohort of children born in large U.S. cities between 1998 and 2000. Participants included 1,647 Black and 689 White young adults who were followed from birth to age 22. Maternal education, household income, family structure, and paternal incarceration were assessed at baseline (birth), and two youth educational outcomes, namely educational attainment and any school disciplinary action, were assessed at age 22 (emerging adulthood). Using structural equation modeling (SEM), mediation analysis was conducted to examine whether household income partially mediates the effects of maternal education on youth educational outcomes, with race (Black vs. White) as the moderator.

**Results:**

The results indicated that maternal education was positively associated with youth educational attainment and negatively associated with school disciplinary actions in the pooled sample that included both Black and White families. However, the effect of parental education on educational attainment at age 22 was weaker for Black than White families. Household income partially mediated racial differences in the effect of maternal education on youth educational attainment. The results suggest that lower household income in Black families is why we observe a weaker effect of parental education on youth educational attainment for Black youth compared to White youth.

**Conclusions:**

Findings suggest that the lower household income of families is one of the reasons high maternal education levels are associated with lower youth educational attainment in Black than White families. Addressing income disparities through tax policies may help reduce racial disparities in education and promote educational equity for Black youth.

## 1. Introduction

Black-White disparities in educational attainment are among the most striking and persistent educational disparities in the United States [1–4]. The Black-White achievement gap reflects disparities in academic performance and outcomes, while the educational attainment gap refers to differences in the level of education attained by race [3, 5–7]. Despite efforts to address these gaps, they have persisted for centuries [3, 6, 8–10]. Understanding the environmental and societal factors that contribute to these disparities is crucial for developing effective interventions and policies to promote educational equity across racial groups [6, 11–15].

Parental education is a critical determinant of offspring educational attainment [16– 22]. Research consistently shows that higher levels of parental education are associated with better educational outcomes for children, including higher academic achievement and graduation rates [17, 19, 23]. This is partly because parents with higher levels of education tend to provide a more stimulating home environment, engage more actively in their children’s education, and have higher expectations for their children’s academic success [24]. Additionally, children with higher parental education have better access to educational resources and attend better schools, which is linked to higher educational aspiration and attainment [25–27]. These factors contribute to the unique role of parental education as a determinant of offspring educational attainment.

Despite the overall positive effects of parental education on educational attainment [27–33], research suggests that this effect may vary by race. Studies have found that the benefits of parental education on offspring health and wellbeing are less pronounced for Black families compared to White families [34, 35]. This is partly because Black individuals with high education often work in lower-paying jobs, resulting in less wealth accumulation [36]. As such, Blacks’ relative disadvantage in the effects of parental education on financial status may contribute to the persistent education gap and achievement gap compared to White individuals, even when there is no racial disparity in parental education [37]. Understanding the factors that underlie this disparity is essential for suggesting policy solutions that may address racial inequalities in educational outcomes.

One area that remains unclear is the role of household income in mediating racial differences in the effects of parental education on educational attainment of offspring between Black and White families [4, 10, 15]. While parental education is an important predictor of offspring educational attainment, household income may also play a significant role [10]. Low-household income families may face additional barriers to the educational success of their offspring, such as limited access to high-quality schools, lack of resources for educational enrichment activities, and greater exposure to economic stressors [15]. These factors may interact to bound the effects of parental education, influencing educational outcomes in complex ways [34, 35]. Understanding the interplay between parental education, household income, and offspring educational outcomes is crucial for developing targeted policy interventions to reduce educational disparities by race [38–41].

### 1.1. Aims and Hypothesis

This paper first aims to explore the role of parental education in shaping the educational outcomes of offspring in Black and White families. Then, we focus on whether household income might mediate Black-White disparities in the effects of parental education on youth educational attainment. Using data from the Fragile Families and Child Wellbeing Study (FFCWS) spanning 22 years, this study provides a unique opportunity to investigate the complex and non-linear interactions between race, parental education, and household income in influencing the educational attainment of children across diverse racial backgrounds. We hypothesized differential effects of parental education on offspring educational attainment, with a relative disadvantage of Black to White families. We then hypothesize that lower household income levels will mediate racial differences in the effects of parental education in Black than White families, thus collectively contributing to the education gap and achievement gap between Black and White youth. This work enables us to study how race, parental education, and household income longitudinally interact and shape offspring educational outcomes over a 22-year period. Our aim is to test whether household income is one of the mechanisms through which parental education generates different educational attainment in youth based on race. The results would inform whether household income redistribution policies based on tax or baby bonds can be useful interventions aimed at reducing the racial gap in youth educational inequalities by promoting the return of parental education in Black families [42, 43].

## 2. Materials and Methods

### 2.1. Design and Setting

The Future of Families and Child Wellbeing Study (FFCWS), formerly known as the Fragile Families and Child Wellbeing Study [44–49], is a comprehensive research project aimed at understanding the challenges faced by economically disadvantaged families in the United States. The FFCWS has followed a birth cohort from 1998, tracking their progress from birth through young adulthood at age 22 in 2022. For detailed information on the sampling techniques and methodology used, please read here [50]. Here, we provide an overview of the FFCWS’s research approach.

### 2.2. Study Institutional Review Board

The study’s protocol was approved by the Institutional Review Board at Princeton University. All participating families gave their informed consent, with parents or legal guardians consenting on behalf of the minors, who also provided their assent. All data collection, storage, and analysis procedures were designed to protect participants’ anonymity, and families were compensated for their participation.

### 2.3. Sample and Sampling

The FFCWS selected a diverse group of urban families across 20 major U.S. cities, each with a population exceeding 200,000. The study focused on underrepresented families, particularly those that were non-married and identified as Black or Hispanics. Consequently, the study’s sample primarily includes families with low socioeconomic status and a significant number of Black and Hispanic participants, which does not reflect the general U.S. population. The analytical sample consisted of 2,336 families with Black or White children with 22 years of data. Families were excluded if they were of Hispanic ethnicity or if the child did not have 22-year data.

### 2.4. Analytical Sample

Our analysis utilized data from the first (years 1998/2000) and seventh (years 2020/2022) waves of the FFCWS. We examined the socioeconomic status (SES) of the families at baseline and the educational outcome of the young adults 22 years later. Our sample included 2,336 Black or White births and their follow up as well as data on their mothers (or caregivers), fathers, and caregivers, over time.

### 2.5. Data Collection and Variables

Data collection involved interviews with both parents, gathering information on various factors including maternal age, child sex, maternal education, and fathers’ history of incarceration. This FFCWS dual-parent data collection approach is critical for ensuring greater accuracy in reporting sensitive information such as incarceration history. Educational attainment, incarceration history, and all other variables were self-reported by the participants at this age. Race and ethnicity were self-identified by the father and the mother. Family structure was measured at the time of birth and was a dichotomous variable: Married vs. any other status.

We assessed primary socioeconomic status (SES) indicators through maternal education at the beginning of the study. Paternal education was not included due to a significant proportion of missing data, attributed to various reasons such as unknown fathers, mothers not in a relationship with the father, or fathers not being part of the study. Therefore, we chose to use maternal education as the predictor, categorized into four levels: (1) less than high school, (2) high school diploma, (3) some college, and (4) college degree or beyond.

Young adult education at age 22 was evaluated using self-reported data. It was categorized into four levels: (1) less than high school, (2) high school graduate, (3) some college, and (4) college graduate. For sensitivity analysis, we also tested the results with years of education (schooling) completed at age 22 and high school dropout as outcomes. However, as the results did not show major changes, we only presented the results of our primary analysis.

Our other outcome was any school disciplinary action against the youth while they were in high school. Disciplinary actions included expulsion, out-of-school suspension, and in-school suspension. We created a dichotomous binary outcome reflecting any disciplinary action versus none. Disciplinary actions were self-reported by the young adults at age 22.

### 2.6. Statistical Analysis

We conducted data analysis using Stata version 18.0. Descriptive statistics, including frequencies (percentages) and means (standard deviations), were provided. Bivariate analysis was performed using the Pearson correlation test. For the multivariable analysis, we employed two structural equation models (SEM) to examine maternal education as the predictor and the household income as the mediator of racial difference in the effects of parental education on offspring educational attainment. These models considered young adult educational attainment and school disciplinary action as the outcomes, race as the moderator, and maternal education as the predictor. In the first model, we did not include household income as the mediator and only controlled for covariates such as child sex, father incarceration, family structure and maternal age at birth. For Model 2, we included our mediator namely household income. Conventional fit indices (Goodness of fit Index (GFI)), including Chi-square divided by degrees of freedom (CMIN/DF), Root Mean Square Error of Approximation (RMSEA), Comparative fit index (CFI) were used to evaluate the goodness of fit.

## 3. Results

### 3.1. Descriptive Data

Participants included 1,647 Black and 689 White young adults who were followed from birth to age 22. As shown in Table 1, the average years of education was 13.3, which was higher for White than Black young adults. Maternal education was higher for White than Black youth. Maternal age was considerably younger for Black than White youth.

### 3.2. Bivariate Correlations

As shown by Table 2, young adult education was positively correlated with family marital status and inversely correlated with Black race. School disciplinary actions were inversely correlated with educational attainment of young adult at age 22.

### 3.3. Multivariable Models

**Figure 1.**
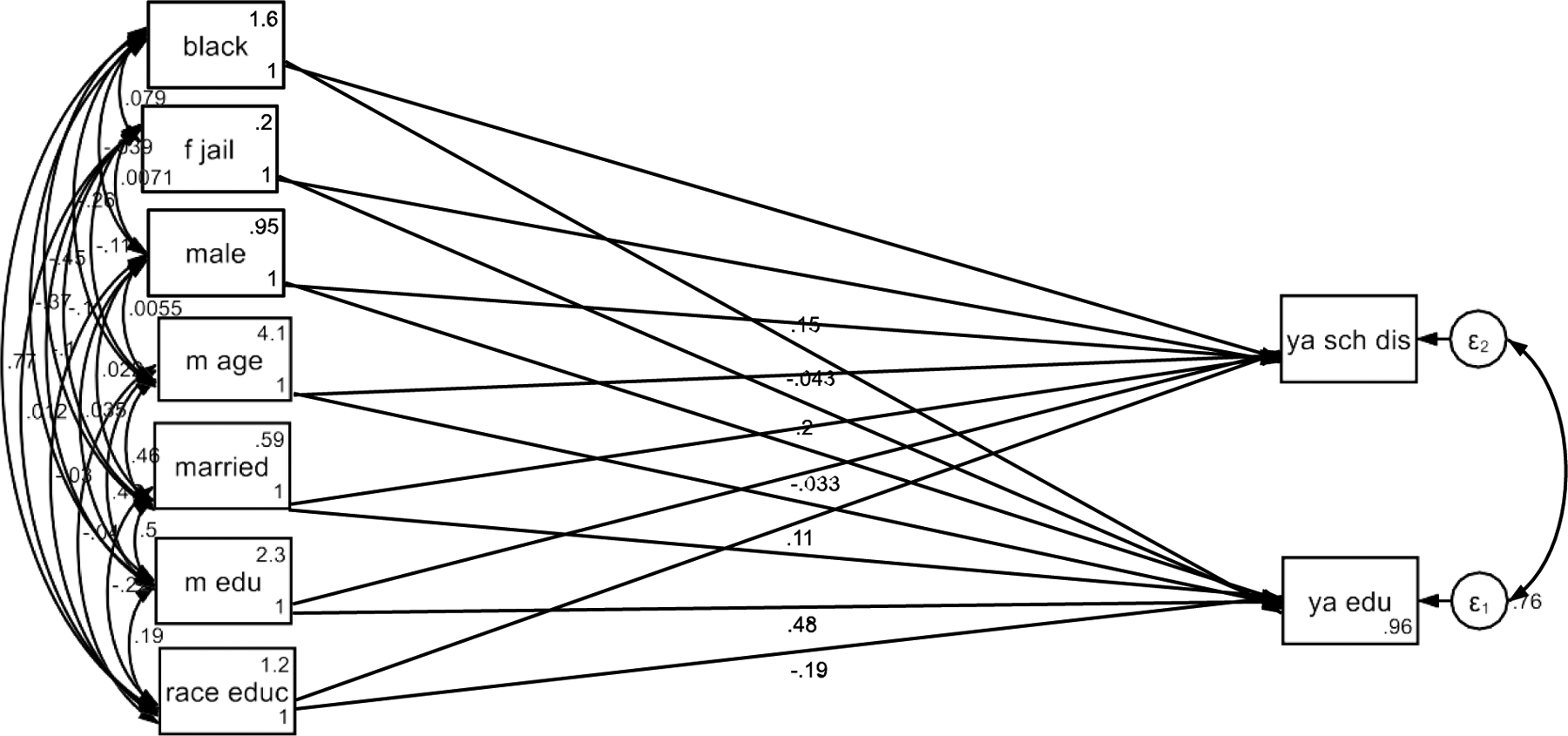
Summary of SEM Without Household Income as the Mediator (HH: Household; YA/EA: Young Adult/Emerging Adult)

As shown in Table 3, the Model 1 showed that maternal education was positively associated with youth education outcomes in the pooled sample that included both Black and White families. However, the effect of maternal education on young adult educational attainment was stronger for White families compared to Black families (Figure 1).

**Figure 2.**
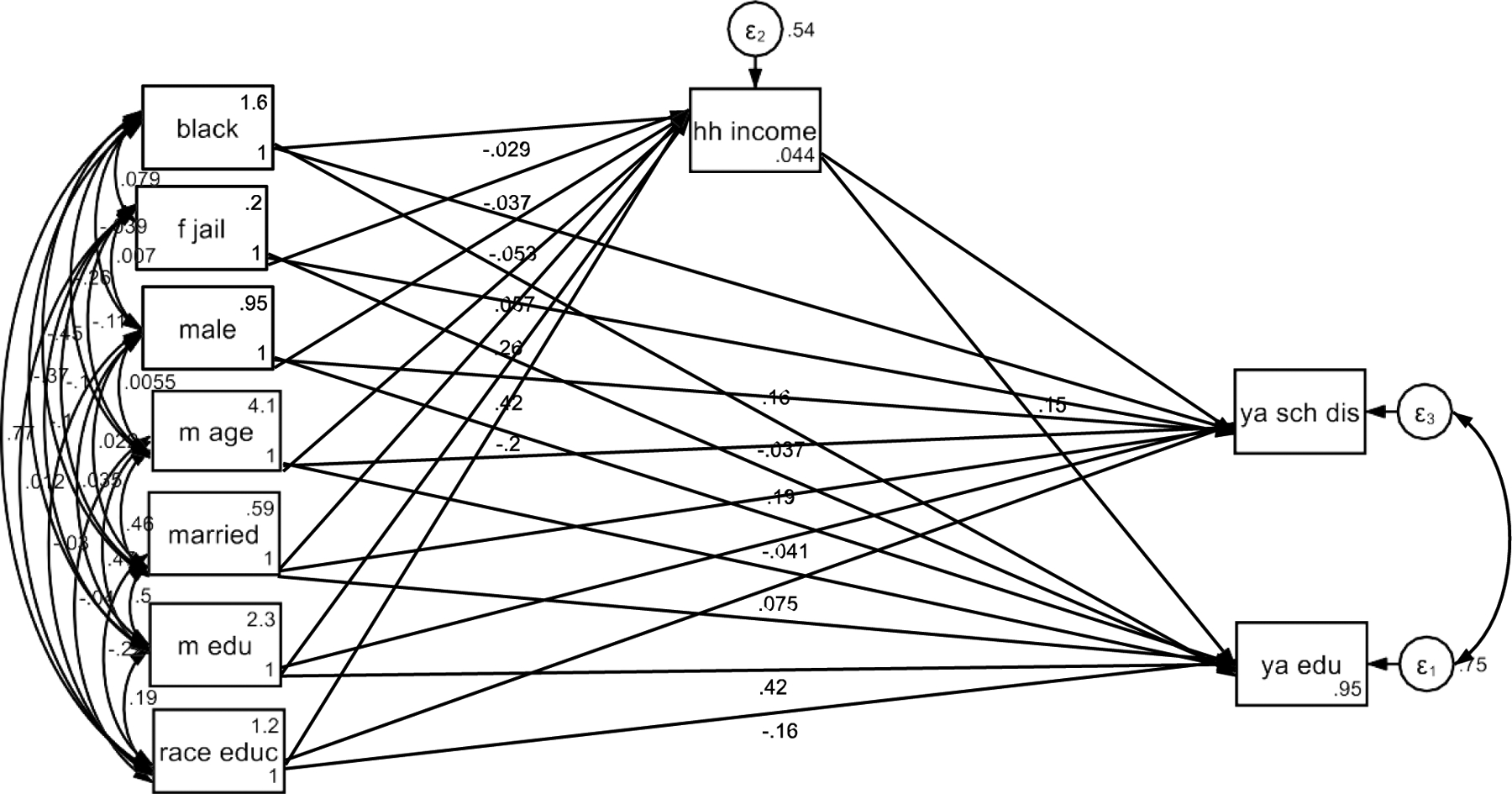
Summary of SEM With HH Income as the Mediator (HH: Household; YA/EA: Young Adult/Emerging Adult)

As shown in Table 4, household income partially mediated the racial differences in the effects of maternal education on youth educational attainment. This means, lower HH is why we observe an attenuated effect of parental education on young adult educational attainment (Figure 2).

## 4. Discussion

This study aimed to compare Black and White families regarding the effects of parental education on offspring educational attainment and school disciplinary actions 22 years later and to investigate the mediating role of household income in explaining the differential effects of maternal education on these educational outcomes between Black and White families. We hypothesized that household income would partially mediate the weaker effects of parental education on offspring educational outcomes, which is crucial for developing targeted interventions to reduce educational inequalities.

First, we found that higher levels of maternal education were associated with better educational outcomes for youth. This effect could be seen for both educational attainment and school disciplinary actions. Second, we found that the effect of maternal education on young adult educational attainment was weaker for Black families than for White families. Third, we observed partial mediation, indicating that household income plays a significant role in mediating racial differences in the effects of maternal education on youth educational attainment. Our results suggest that lower household income in Black families is why we observe a weaker effect of parental education on youth educational attainment for Black youth compared to White youth. These findings highlight the complex interplay between parental education, household income, and race in shaping educational outcomes for youth.

Parental education has long been recognized as a critical determinant of child development, including educational outcomes [22, 34, 51–55]. Parents with higher levels of education tend to provide a more stimulating home environment, engage more actively in their children’s education, and have higher expectations for their children’s academic success [56–58]. These factors contribute to the positive association between parental education and child educational attainment. However, our findings suggest that the benefits of parental education may be attenuated for Black families compared to White families, highlighting the need for targeted interventions to support educational success in Black communities [59, 60].

Household income is another important factor influencing children’s educational success. Higher income levels are associated with access to better schools, educational resources, and opportunities for enrichment activities. Additionally, higher household income can reduce financial stressors within the household, which can positively impact children’s educational outcomes. Our findings suggest that household income plays a significant mediating role in the effects of maternal education on youth educational attainment, particularly in Black families. This underscores the importance of addressing household income disparities as a means of promoting educational equity.

Despite the positive effects of parental education and household income on children’s educational outcomes, Black communities often face lower education opportunities compared to White communities. Structural barriers such as unequal access to quality education, economic disparities, and systemic racism contribute to these inequalities. There is a need for policies that address these disparities and promote educational opportunities in Black communities.

The concept of Minorities’ Diminished Returns (MDRs) [61, 62] suggests that the beneficial effects of socioeconomic resources, such as parental education and household income, are weaker for minority populations compared to White populations. This phenomenon may help explain why the effect of maternal education was stronger in White than Black families [63]. These diminished returns underscore the importance of addressing the unique challenges faced by minority populations and the need for structural interventions that account for these disparities.

Previous research has shown that the positive effects of parental education on household income, poverty, and wealth are weaker for Black individuals compared to White individuals [36, 62, 64, 65]. This suggests that the intergenerational transmission of socioeconomic status is less effective in Black families, contributing to the persistence of racial disparities in household income and wealth [66, 67]. Our findings support the notion of MDRs and highlight the need for bold innovative policies that address the systemic barriers faced by Black families and communities in achieving economic and educational success [61].

### 4.1. Policy Implications

Addressing the educational inequalities highlighted in this study requires a multifaceted multisector policy approach [61, 68, 69]. Economic, social, and public policies that aim at reducing household income disparities, increasing access to quality education in Black communities, and addressing systemic racism are essential for promoting educational equity [70, 71]. Our findings underscore the importance of targeted interventions that consider the unique needs and challenges faced by Black families and communities in capitalizing their education and achieving economic and educational success.

### 4.2. Future Research Directions

Future research should continue to explore the mechanisms such as household income that explain differential influence of parental education on outcomes of youth in Black and White families. Longitudinal studies that follow children from early childhood into adulthood can provide valuable insights into the long-term effects of parental education and household income on educational attainment and economic mobility [72]. Additionally, research that examines the intersectionality of race, gender, sex, income, and other factors on educational outcomes can further our understanding of the complex dynamics at play [73–76].

### 4.3. Limitations and Strengths

This study has several limitations, including omitted variables such as school quality, paternal education, and wealth, which limits our ability to draw causal conclusions. Additionally, the sample was drawn from a specific cohort of families, which may limit the generalizability of the findings. The sample was not random, and longitudinal cohort studies such as the FFCWS have high attrition rates. However, the use of longitudinal data and the inclusion of a diverse sample of families are strengths of this study. Future research should aim to address these limitations by using longitudinal data and diverse samples to provide a more comprehensive understanding of the factors influencing educational outcomes in Black and White families.

## 5. Conclusion

In conclusion, this study provides evidence for the mediating role of household income in explaining the weaker effects of maternal education on youth educational attainment in Black than White families. Addressing household income disparities and structural barriers is crucial for promoting educational equity and reducing racial disparities in educational outcomes. Our findings highlight the importance of considering the complex interplay between parental education, household income, and race in shaping educational outcomes for youth, and underscore the need for targeted interventions to address these disparities.

## Figures and Tables

**Table 1.** Descriptive Data Overall and by Race

	All N=2,336		White N=689		Black N=1,647	
	Mean	Std. Err.	Mean	Std. Err.	Mean	Std. Err.
Maternal Education (1–4)	2.32	0.02	2.78	0.04	2.09	0.03
Maternal Age (Years)	25.43	0.15	27.52	0.27	24.39	0.17
YA Education (Years)	13.33	0.03	13.59	0.06	13.20	0.04
HH Income	3.53	0.08	6.12	0.18	2.51	0.06

YA = Young Adults; HH = Household

**Table 2.** Bivariate Correlations

	1	2	3	4	5	6	7
1 Race (Black)	1.00						
2 Maternal Education (Baseline)	−0.37*	1.00					
3 Father Jailed (Baseline)	0.08*	−0.10*	1.00				
4 HH Married (Baseline)	−0.45*	0.51*	−0.10*	1.00			
5 YA Educational Attainment (At Age 22)	−0.21*	0.43*	−0.09*	0.32*	1.00		
6 YA School Discipline, Any (Baseline)	0.31*	−0.30*	0.11*	−0.27*	−0.36*	1.00	
7 Sex (Male)	−0.04	0.04	0.01	0.02	−0.18*	0.13*	1.00

* p < 0.05 (Pearson); HH: Household; YA/EA: Young Adult/Emerging Adult

**Table 3.** Structural Equation Model (SEM) Without Household Income as the Mediator

		Standardized Coefficient	SE.	95%	CI	p
YA Educational Attainment (At Age 22)	Maternal Age (Baseline)	−0.03	0.02	−0.08	0.01	0.173
	Maternal Education (Baseline)	0.48	0.04	0.40	0.56	< 0.001
	Father Jailed (Baseline)	−0.04	0.02	−0.08	0.00	0.037
	Sex (Male)	−0.20	0.02	−0.24	−0.16	< 0.001
	Race (Black)	0.15	0.06	0.04	0.26	0.008
	Married HH (Baseline)	0.11	0.03	0.06	0.16	< 0.001
	Race (Black) × Maternal Education (Baseline)	−0.19	0.06	−0.29	−0.08	0.001
	Intercept	0.96	0.14	0.69	1.23	< 0.001
YA Any School Discipline (At Age 22)	Maternal Age (Baseline)	−0.07	0.03	−0.12	−0.02	0.003
	Maternal Education (Baseline)	−0.19	0.04	−0.27	−0.10	< 0.001
	Father Jailed (Baseline)	0.06	0.02	0.02	0.11	0.003
	Sex (Male)	0.14	0.02	0.10	0.19	< 0.001
	Race (Black)	0.16	0.06	0.04	0.28	0.007
	Married HH (Baseline)	−0.05	0.03	−0.10	0.01	0.078
	Race (Black) × Maternal Education (Baseline)	0.05	0.06	−0.06	0.16	0.388

* p < 0.05; HH: Household; YA/EA: Young Adult/Emerging Adult

**Table 4.** Structural Equation Model (SEM) With Household Income as the Mediator

		Standardized Coefficient	SE.	95%	CI	p
YA Educational Attainment (At Age 22)	HH Income (Baseline)	0.15	0.03	0.09	0.20	< 0.001
	Maternal Age (Baseline)	−0.04	0.02	−0.09	0.01	0.085
	Maternal Education (Baseline)	0.42	0.04	0.33	0.50	< 0.001
	Father Jailed (Baseline)	−0.04	0.02	−0.08	0.00	0.067
	Race (Black) × Maternal Education (Baseline)	−0.16	0.06	−0.26	−0.05	0.004
	Sex (Male)	−0.19	0.02	−0.23	−0.15	< 0.001
	Race (Black)	0.16	0.06	0.05	0.27	0.006
	Married HH (Baseline)	0.08	0.03	0.02	0.13	0.005
	Intercept	0.95	0.14	0.69	1.22	< 0.001
HH Income (Baseline)	Maternal Age (Baseline)	0.06	0.02	0.02	0.10	0.005
	Maternal Education (Baseline)	0.42	0.03	0.35	0.49	< 0.001
	Father Jailed (Baseline)	−0.04	0.02	−0.07	0.00	0.033
	Race (Black) × Maternal Education (Baseline)	−0.20	0.05	−0.30	−0.11	< 0.001
	Sex (Male)	−0.05	0.02	−0.09	−0.02	0.002
	Race (Black)	−0.03	0.05	−0.12	0.07	0.545
	Married HH (Baseline)	0.26	0.02	0.21	0.30	< 0.001
	Intercept	0.04	0.11	−0.18	0.27	0.697
	HH Income (Baseline)	−0.07	0.03	−0.13	−0.02	0.010
YA Any School Discipline (At Age 22)	Maternal Age (Baseline)	−0.07	0.03	−0.12	−0.02	0.006
	Maternal Education (Baseline)	−0.16	0.04	−0.25	−0.07	< 0.001
	Father Jailed (Baseline)	0.06	0.02	0.02	0.10	0.004
	Race (Black) × Maternal Education (Baseline)	0.03	0.06	−0.08	0.15	0.548
	Sex (Male)	0.14	0.02	0.10	0.18	< 0.001
	Race (Black)	0.16	0.06	0.04	0.28	0.008
	Married HH (Baseline)	−0.03	0.03	−0.08	0.03	0.298
	Intercept	1.30	0.14	1.02	1.57	< 0.001

HH: Household; YA/EA: Young Adult/Emerging Adult

## Data Availability

FFCWS data are available to public at Office of Population Research data repository available at https://oprdata.princeton.edu.
